# External Validation of the Walter Index for Posthospitalization Mortality Prediction in Older Adults

**DOI:** 10.1001/jamanetworkopen.2024.55475

**Published:** 2025-01-22

**Authors:** Thiago J. Avelino-Silva, Sei J. Lee, Kenneth E. Covinsky, Louise C. Walter, W. James Deardorff, John Boscardin, Flavia Campora, Claudia Szlejf, Claudia K. Suemoto, Alexander K. Smith

**Affiliations:** 1 Division of Geriatrics, School of Medicine, University of California San Francisco; 2 Laboratorio de Investigacao Medica em Envelhecimento (LIM-66), Servico de Geriatria, Hospital das Clinicas (HCFMUSP), Faculdade de Medicina, Universidade de São Paulo, São Paulo, Brazil; 3 Geriatrics, Palliative and Extended Care Service Line, San Francisco Veterans Administration Health Care System, San Francisco, California; 4 Hospital Israelita Albert Einstein, São Paulo, Brazil

## Abstract

**Importance:**

The Walter Index is a widely used prognostic tool for assessing 12-month mortality risk among hospitalized older adults. Developed in the US in 2001, its accuracy in contemporary non-US contexts is unclear.

**Objective:**

To evaluate the external validity of the Walter Index in predicting posthospitalization mortality risk in Brazilian older adult inpatients.

**Design, Setting, and Participants:**

This prognostic study used data from a cohort of adults aged 70 years or older admitted to the geriatric unit of a university hospital in Brazil from January 1, 2009, to February 28, 2020. Participants underwent comprehensive geriatric assessments at admission, were reevaluated at discharge, and were subsequently followed up for 48 months. Data were analyzed from March to July 2024.

**Main Outcomes and Measures:**

The Walter Index, a score based on 6 risk factors (male sex, dependent activities of daily living at discharge, heart failure, cancer, high creatinine level, and low albumin level), was calculated to assess its predictive accuracy for 12-month mortality as well as 6-, 24-, and 48-month mortality. The study investigated whether incorporating delirium, frailty, or C-reactive protein level enhanced accuracy. Performance was assessed using discrimination, calibration, and clinical utility measures.

**Results:**

In total, 2780 participants (mean [SD] age, 81 [7] years; 1795 [65%] female) were included, with 89 (3%) lost to follow-up. The 12-month posthospitalization mortality rate was 23% (646 participants). Mortality was 7% (47 of 634) in the lowest-risk group (0-1 point), 17% (111 of 668) for 2 to 3 points, 25% (198 of 803) for 4 to 6 points, and 43% (290 of 675) in the highest-risk group (≥7 points). The index demonstrated an area under the receiver operating characteristic curve (AUC) of 0.714 (95% CI, 0.691-0.736) for predicting 12-month posthospitalization mortality (AUCs were 0.75 and 0.80 in the original derivation and validation cohorts, respectively). Comparable results were observed for mortality at 6 months (AUC, 0.726; 95% CI, 0.700-0.752), 24 months (AUC, 0.711; 95% CI, 0.691-0.730), and 48 months (AUC, 0.719; 95% CI, 0.700-0.738). Adding delirium modestly increased the index’s discrimination (AUC, 0.723; 95% CI, 0.702-0.749); additionally including frailty and C-reactive protein level did not improve discrimination further (AUC, 0.723; 95% CI, 0.701-0.744).

**Conclusions and Relevance:**

In this prognostic study of hospitalized older adults in Brazil, the Walter Index showed similar discrimination in predicting postdischarge mortality as it did 2 decades ago in the US. These findings highlight the need for continuous validation and potential modification of established prognostic tools to improve their applicability across settings.

## Key Points

**Question:**

Does the Walter Index, developed in the US to assess 12-month mortality risk among hospitalized older adults, predict posthospitalization mortality in older adults outside the US?

**Findings:**

In this prognostic study of 2780 adults aged 70 years or older in Brazil, the Walter Index demonstrated predictive accuracy for 12-month mortality comparable to that in the original US cohort.

**Meaning:**

In this study, the Walter Index demonstrated strong transportability and generalizability in predicting mortality in a contemporary non-US setting.

## Teaser

This prognostic study investigates whether the Walter Index, developed to assess 12-month mortality risk in US older adults after hospitalization, has comparable predictive accuracy among older adults in Brazil.

## Introduction

Accurate prognostic assessments targeting the posthospitalization period are vital for older adults, guiding clinicians and families in making informed decisions that extend beyond immediate medical care.^1^ These assessments help tailor care plans to align with each patient’s prognosis and preferences.^2,3^ While several tools have been developed to predict posthospitalization mortality in older adults, many external validation studies have had small sample sizes, nonrepresentative datasets, and improper evaluation methods.^4,5,6^

The Walter Index, published in 2001, stands out as a widely used prognostic tool that predicts 12-month mortality following hospitalization in patients aged 70 years or older.^7^ Developed in a cohort of 1495 US patients and validated in a separate cohort of 1427 US patients, it is the most used inpatient score on ePrognosis, a repository of published prognostic indexes for older adults.^8^ The Walter Index supports clinical decision-making, enhances communication with patients and families, and informs discharge care plans.^1,9,10,11,12,13,14^ However, its performance in low- and middle-income countries like Brazil, which has the second-largest population in the western hemisphere and is the second-largest user of ePrognosis, remains largely unexplored. This lack of validation challenges several aspects of transportability across different health care systems, cultures, and periods, possibly impacting the tool’s accuracy.^15,16,17,18^

Identifying vulnerable patients for targeted interventions is essential to mitigate adverse outcomes, especially in underserved populations.^19^ In lower-income countries, hospital-based geriatric screenings have effectively reduced mortality rates and readmissions.^20^ Established prognostic tools like the Charlson Comorbidity Index have shown varying levels of effectiveness in predicting outcomes such as mortality, institutionalization, and readmissions.^1,14,20,21,22,23^ However, integration into routine hospital protocols is limited due to miscalibrated scores and cultural differences.^20,24^ Therefore, systematic validation and context-specific adaptation of prognostic tools are necessary to ensure their effectiveness.

Our study addressed this gap by assessing the external validity of the Walter Index in a large cohort of non-US hospitalized older adults. Furthermore, we investigated whether including delirium, frailty, and C-reactive protein (CRP) levels—risk factors for mortality that were unavailable at the time when the original index was developed—could enhance its predictive accuracy.

## Methods

### Study Design and Setting

This prospective prognostic study was conducted at Hospital das Clinicas, University of São Paulo Medical School (HCFMUSP), a 2200-bed public tertiary university hospital in Brazil. We recruited participants at the HCFMUSP geriatric unit, which primarily admits nonsurgical, nonorthopedic patients aged 60 years or older. The study adhered to the Transparent Reporting of a Multivariable Prediction Model for Individual Prognosis or Diagnosis (TRIPOD+AI) guidelines and received approval from the HCFMUSP institutional review board.^25^ Written informed consent was obtained from all participants or their surrogates before data collection. No compensation was provided for participation.

### Participant Recruitment

We used data from discharged participants consecutively admitted to the geriatric unit from January 1, 2009, to February 28, 2020. Exclusion criteria included age less than 70 years and a hospital stay shorter than 2 days, aligning with the parameters used in the original development of the Walter Index.

### Data Collection and Assessment Protocols

A standardized geriatric assessment protocol was completed for each participant within 24 hours of admission and was repeated at discharge.^21^ The assessment covered demographics (eg, age, sex, self-reported race and ethnicity, and educational attainment), medical history (eg, number of medications, comorbidities, and frailty), cognitive and functional capabilities (eg, dementia per the Clinical Dementia Rating, delirium, and activities of daily living [ADLs]), and initial laboratory values (eg, CRP, creatinine, and albumin levels). Race and ethnicity data were included to characterize the diversity of the sample; categories were Black, White, and other (Asian, Indigenous, or not otherwise specified). Hospitalization details and outcomes were documented at discharge. Further details on our assessment protocols can be found in previous work.^21,26,27^

The Walter Index estimates mortality risk within 1 year following hospital discharge, assigning scores ranging from 0 to 20 (higher scores indicate higher mortality risk) based on 6 risk factors: male sex (1 point), dependency in ADLs at discharge (1-4 dependencies, 2 points; 5 dependencies, 5 points), existing congestive heart failure (2 points), cancer status (solitary, 3 points; metastatic, 8 points), elevated creatinine level (>3.0 mg/dL, 2 points), and reduced albumin level (3.0-3.4 g/dL, 1 point; <3.0 g/dL, 2 points) (to convert creatinine to μmol/L, multiply by 88.4; to convert albumin to g/L, multiply by 10). Consistent with the original Walter Index, we assessed functional status at discharge using a modified version of the Katz Index of ADLs.^28^ We evaluated independence in bathing, dressing, toileting, transferring, and eating, with dependency determined based on whether personal assistance was required for each activity. Similarly, following the strategy used by Walter et al,^7^ we used creatinine and albumin levels obtained at admission, as discharge laboratory values were not always routinely available.

In addition to the Walter Index components, delirium, frailty, and CRP level were assessed to explore whether they could improve the tool. These factors were selected based on their established roles as significant predictors of mortality in older adults and their growing prominence in contemporary geriatric care.^16,17,29^ Delirium and frailty are common geriatric syndromes, and research in the past 2 decades has solidified these conditions as important predictors of mortality. C-reactive protein is a widely recognized biomarker of systemic inflammation that has been linked to higher mortality. While other risk factors might be justifiable alternatives, delirium, frailty, and CRP level were chosen due to their relevance and availability in clinical practice. By focusing on factors that are routinely available, our hope was that a modified Walter Index would be easy to implement in routine care.

Delirium was diagnosed using the Confusion Assessment Method, which was administered daily throughout the hospital stay. This method is a structured tool that helps identify delirium by assessing acute changes and fluctuations in mental status, inattention, and either disorganized thinking or altered level of consciousness.^30^ Any occurrence of delirium during hospitalization was included in our analyses. Frailty was assessed at admission using the FRAIL scale, a simple questionnaire designed to evaluate 5 key factors (fatigue, resistance, ambulation, illnesses, and weight loss), with higher scores indicating greater frailty.^31^ To capture baseline frailty prior to hospitalization, participants were asked to report their symptoms as experienced during the month before admission. The Charlson Comorbidity Index, a widely used prognostic score that predicts mortality by categorizing and assigning weights to various comorbid conditions, was calculated using updated weights as recommended in the literature.^32^ The weights were applied based on all available information from the hospital stay to ensure optimal scoring.

### Outcome Measurement and Follow-Up

Our primary outcome was mortality within 12 months following hospital discharge, and we also explored 6-, 24-, and 48-month mortality. Posthospitalization mortality data were collected using follow-up telephone calls, which included up to 3 attempts to contact patients or their caregivers on different days, including Saturdays. Research assistants conducting the follow-up calls were blinded to the baseline data. In cases in which direct contact was unsuccessful, we reviewed medical records and public registry checks to verify mortality status or determine the last known survival date. Participants without information extending to the end of the follow-up period were classified as lost to follow-up. For survival analyses, these participants were censored at their last known date of survival, and they were excluded from analyses of mortality outcomes based on logistic regressions.

### Statistical Analysis

Baseline characteristics were stratified by 12-month survival status. Continuous variables were reported as means with SDs or medians with IQRs, depending on data distribution, while categorical variables were presented as frequencies and percentages. Comparisons between living participants and those who died used *t* tests or Wilcoxon rank sum tests for continuous variables and χ^2^ or Fisher exact tests for categorical variables. Additionally, we described the cohort’s demographics and mortality rates in relation to the original Walter Index cohort.

Patients were stratified into risk classes based on their Walter Index score (≤1, 2-3, 4-6, or ≥7 points), with corresponding mortality rates and 95% CIs. We conducted a Kaplan-Meier survival analysis to assess the time-to-event data for each stratified group. Cox proportional hazards regression models assessed the association between the Walter Index scores (both total and within specific risk classes) and 12-month mortality. Model discrimination was evaluated using receiver operating characteristic (ROC) curves, and areas under the ROC curve (AUCs) were computed to determine how effectively the Walter Index differentiated between patients who died and patients alive at different time points. Calibration was assessed using calibration plots comparing the predicted mortality probabilities with the observed outcomes.

Next, hazard ratios (HRs) and AUCs were calculated for a model in which the points system was recalibrated to match the specific regression coefficients derived from the cohort. Subgroup analyses according to self-reported race and ethnicity were performed, and additional risk factors—delirium, frailty, and CRP level—were included to evaluate their impact on improving the Walter Index. For each modification in the Walter Index (ie, adding delirium, frailty, or CRP level and adding all 3 factors), we first obtained the initial C statistics and then generated 100 bootstrapped datasets. We performed logistic regressions on each bootstrapped dataset to calculate their C statistics and estimated the optimism as the mean difference between the bootstrapped and original C statistics. This optimism estimate was then used to correct the initial C statistic. To compare the discriminative ability between subgroups and between different versions of the Walter Index, we used tests of equality of AUCs. In addition, to evaluate the clinical utility of the Walter Index in comparison with another commonly used prognostic instrument, we used decision curve analysis to compare the Walter Index with the Charlson Comorbidity Index, assessing the net benefit of using the 2 indexes across various decision thresholds.

Data were analyzed from March to July 2024 using Stata, version 17.0 (StataCorp LLC). A 2-tailed type I error of 5% was considered statistically significant for all tests. We used a prospective 10-year dataset with minimal missing data, opting for a complete case analysis. The cohort exceeded typical thresholds for model validation in the number of both participants and events.^6^

## Results

We included 2780 older adults, of whom 646 (23%) died within 12 months after hospitalization (data were missing for 51 cases [2%]). Sixteen individuals (<1%) were lost to follow-up at 12 months, and a total of 89 individuals (3%) were lost to follow-up over 48 months. The median length of stay at 12 months was shorter for participants alive at 12 months (11 days [IQR, 6-18 days]) compared with those who died (16 days [IQR, 9-27 days]) (*P* < .001). The mean (SD) age of participants was 81 (7) years; 1795 (65%) were female, and 985 (35%) were male. A total of 627 (23%) were Black; 2031 (73%), White; and 122 (4%), other race and ethnicity. At discharge, 1126 participants (41%) were independent in all ADLs, 864 (31%) were dependent in 1 to 4 ADLs, and 790 (28%) were dependent in all ADLs. Individuals who died were significantly older than patients alive at 12 months and had greater ADL dependencies, more geriatric syndromes (eg, frailty, dementia, depression, and polypharmacy), and poorer nutritional status (Table 1).

**Table 1.** Baseline Characteristics of Participants by 12-Month Mortality

Characteristic	Participants^a^			*P* value
	Total (N = 2780)	Alive at 12 mo (n = 2134)	Died by 12 mo (n = 646)	
**Demographics**				
Age, mean (SD), y	81 (7)	81 (6)	83 (7)	<.001
Sex				
Female	1795 (65)	1416 (66)	379 (59)	<.001
Male	985 (35)	718 (34)	267 (41)	
Race and ethnicity				
Black	627 (23)	484 (23)	143 (22)	.95
White	2031 (73)	1556 (73)	475 (74)	
Other^b^	122 (4)	94 (4)	28 (4)	
Educational level, y				
<4	789 (28)	608 (28)	181 (28)	.21
3-7	978 (35)	766 (36)	212 (33)	
≥8	1013 (36)	760 (36)	253 (39)	
**Medical history**				
Medications, median (IQR), No.	6 (4-9)	6 (4-9)	6 (3-9)	.005
Frailty				
Robustness	298 (11)	269 (13)	29 (4)	<.001
Prefrailty	1371 (49)	1091 (51)	280 (43)	
Frailty	1111 (40)	774 (36)	337 (52)	
Depression	919 (33)	732 (34)	187 (29)	.011
Dementia				
Absent	1533 (55)	1268 (59)	265 (41)	<.001
Mild	533 (19)	400 (19)	133 (21)	
Moderate	266 (10)	182 (9)	84 (13)	
Severe	448 (16)	284 (13)	164 (25)	
Hypertension	1986 (71)	1533 (72)	453 (70)	.40
Diabetes	1016 (37)	782 (37)	234 (36)	.85
Stroke	628 (23)	456 (21)	172 (27)	.005
Heart failure	797 (29)	565 (26)	232 (36)	<.001
Coronary disease	572 (21)	432 (20)	140 (22)	.43
COPD	320 (12)	217 (10)	103 (16)	<.001
Cancer				
Absent	2334 (84)	1841 (86)	493 (76)	<.001
Nonmetastatic	332 (12)	248 (12)	84 (13)	
Metastatic	114 (4)	45 (2)	69 (11)	
Charlson Comorbidity Index, median (IQR)	3 (2-5)	3 (2-5)	4 (2-6)	<.001
**At admission**				
Independent ADLs				
Bathing	1351 (49)	1162 (54)	189 (29)	<.001
Dressing	1380 (50)	1193 (56)	187 (29)	<.001
Toileting	1537 (55)	1308 (61)	229 (35)	<.001
Transferring	1552 (56)	1322 (62)	230 (36)	<.001
Eating	1864 (67)	1548 (73)	316 (49)	<.001
Delirium	481 (17)	295 (14)	186 (29)	<.001
CRP level, median (IQR), mg/L	14 (3-47)	11 (3-38)	27 (9-75)	<.001
Creatinine level, median (IQR), mg/dL	1.0 (0.8-1.4)	1.0 (0.8-1.3)	1.1 (0.8-1.6)	<.001
Albumin level, median (IQR), g/dL	3.6 (3.1-4)	3.7 (3.2-4.1)	3.3 (2.9-3.7)	<.001
**At discharge**				
ADLs (independent)				
Bathing	1807 (65)	1484 (70)	323 (50)	<.001
Dressing	1789 (64)	1473 (69)	316 (49)	<.001
Toileting	1968 (71)	1597 (75)	371 (57)	<.001
Transferring	2010 (72)	1622 (76)	388 (60)	<.001
Eating	2182 (78)	1757 (82)	425 (66)	<.001
Delirium^c^	823 (30)	514 (24)	309 (48)	<.001
Walter Index risk group^d^				
≤1 point	634 (23)	587 (28)	47 (7)	<.001
2-3 points	668 (24)	557 (26)	111 (17)	
4-6 points	803 (29)	605 (28)	198 (31)	
≥7 points	675 (24)	385 (18)	290 (45)	

Abbreviations: ADLs, activities of daily living; COPD, chronic obstructive pulmonary disease; CRP, C-reactive protein.

SI conversion factors: To convert creatinine to μmol/L, multiply by 88.4; CRP to mg/L, multiply by 10; albumin to g/L, multiply by 10.

^a^ Data are presented as number (percentage) of participants unless otherwise indicated.

^b^ Other includes Asian, Indigenous, or not otherwise specified.

^c^ Includes delirium at admission and incident delirium during hospitalization.

^d^ Score range, 0 to 20, with higher scores indicating higher mortality risk.

**Figure 1.**
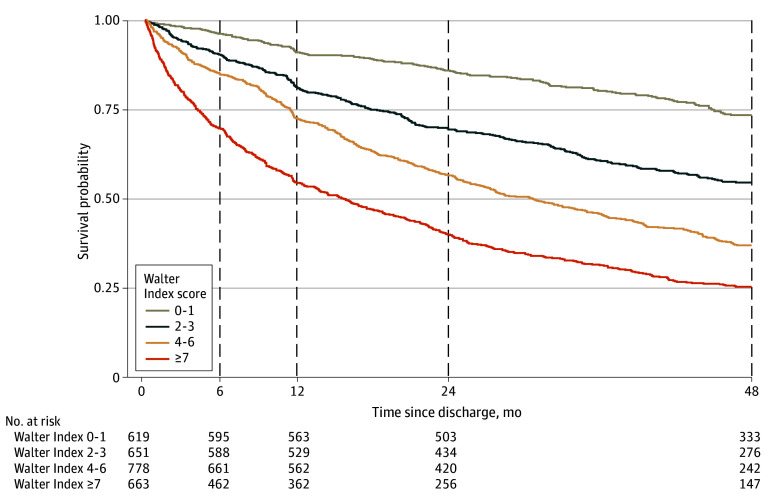
Kaplan-Meier Survival Estimates at 6, 12, 24, and 48 Months According to Walter Index Risk Groups

Among patients alive at 12 months, the median Walter Index score was 3 (IQR, 1-6), while for those who died, the median score was 6 (IQR, 4-8). Stratification by Walter Index scores revealed significant differences in survival probabilities across risk groups, as confirmed by the log-rank test (Table 2 and Figure 1). Posthospitalization mortality rates were 14% (398 of 2780 participants) at 6 months, 36% (999 of 2780) at 24 months, and 50% (1396 of 2780) at 48 months. Mortality was 7% (47 of 634) in the lowest-risk group (0-1 point), 17% (111 of 668) for 2 to 3 points, 25% (198 of 803) for 4 to 6 points, and 43% (290 of 675) in the highest-risk group (≥7 points). There were distinct survival curves according to Walter Index risk groups (Figure 1).

**Table 2.** Characteristics and Predictive Performance of the Walter Index in the Original and External Validation Cohorts

Measure	Walter Index, 2001		Walter Index, external validation	
	Derivation cohort	Validation cohort	Not recalibrated	Recalibrated^a^
12-mo Mortality, % (95% CI)				
Group 1	13 (10-16)	4 (2-6)	7 (6-10)	8 (6-10)
Group 2	20 (16-24)	19 (15-23)	17 (14-20)	20 (17-23)
Group 3	37 (33-41)	34 (29-39)	25 (22-28)	28 (25-32)
Group 4	68 (63-73)	64 (58-70)	43 (39-47)	47 (43-51)
AUC (95% CI)	0.75 (NA)	0.80 (NA)	0.714 (0.691-0.736)	0.716 (0.694-0.738)

Abbreviations: AUC, area under the receiver operating characteristic curve; NA, not available.

^a^ Original risk groups were classified by Walter Index risk score (range, 0-20 points, with higher scores indicating higher mortality risk) as follows: group 1 (≤1 point), group 2 (2-3 points), group 3 (4-6 points), and group 4 (≥7 points). The recalibrated risk groups were as follows: group 1 (≤2 points), group 2 (3-5 points), group 3 (6-8 points), and group 4 (≥9 points). The recalibration was performed using logistic regression analysis, incorporating the original Walter Index components into the model and adjusting the scores based on the coefficients derived from the external validation cohort data. The test for equality of AUCs comparing the recalibrated and not recalibrated models had a *P* value of .61.

**Figure 2.**
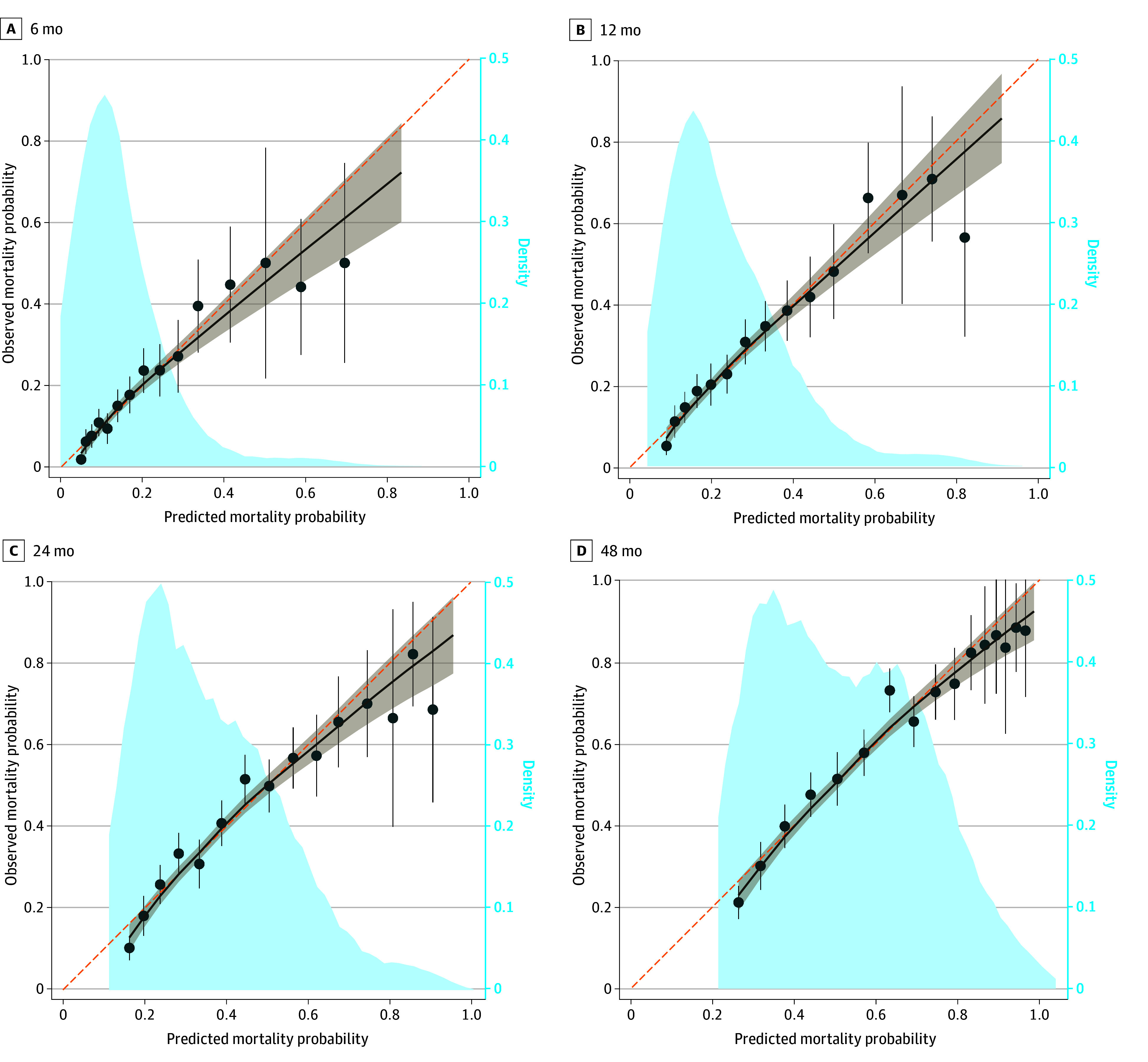
Calibration Plots for the Walter Index External Validation Data markers show Walter Index predicted mortality probabilities, and error bars represent 95% CIs. Dashed diagonal lines indicate the ideal calibration, where predicted probabilities perfectly match observed outcomes, and solid lines indicate the smoothed calibration curves, with the tan shaded area representing 95% CIs. Light blue shaded areas represent the density of predicted mortality probabilities, with higher peaks reflecting a higher concentration of predictions.

Each additional point on the Walter Index was associated with an increase in the HR for 12-month mortality (HR, 1.19; 95% CI, 1.17-1.22), with little change after recalibrating scores to our cohort-specific coefficients (HR, 1.17; 95% CI, 1.15-1.20). The discriminative ability and calibration of the index are illustrated by ROC curves (eFigure 1 in Supplement 1) and calibration plots (Figure 2).

The Walter Index AUC was lower than in the original study^7^ (AUCs of 0.75 and 0.80 in the derivation and validation cohorts, respectively) at 0.714 (95% CI, 0.691-0.736) and did not change significantly when scores were recalibrated to our cohort-specific coefficients (Table 2 and eTable 1 in Supplement 1). Subgroup analysis showed no significant variation in the predictive performance of the Walter Index across different racial and ethnic groups. Among participants who were not White, the AUC was 0.717 (95% CI, 0.675-0.760), and for White participants, it was 0.713 (95% CI, 0.687-0.738) (*P* = .85). The AUCs for predicting 6-, 24-, and 48-month mortality were 0.726 (95% CI, 0.700-0.752), 0.711 (95% CI, 0.691-0.730), and 0.719 (95% CI, 0.700-0.738), respectively.

The Walter Index combined with delirium alone showed an optimism-corrected AUC of 0.723 (95% CI, 0.702-0.749); adding only frailty yielded an AUC of 0.713 (95% CI, 0.690-0.736), and adding only CRP level yielded an AUC of 0.716 (95% CI, 0.694-0.743) (Table 3). Adding the 3 new factors simultaneously did not improve discrimination further compared with adding delirium alone (AUC, 0.723; 95% CI, 0.701-0.744; *P* = .10).

**Table 3.** Comparison of Modified Versions of the Walter Index in the External Validation Cohort

Measure	Walter Index plus delirium	Walter Index plus frailty	Walter Index plus CRP	Walter Index plus delirium, frailty, and CRP^a^
Apparent AUC (95% CI)	0.726 (0.704-0.748)	0.715 (0.693-0.737)	0.718 (0.696-0.740)	0.728 (0.706-0.750)
Optimism-corrected AUC (95% CI)^b^	0.723 (0.702-0.749)	0.713 (0.690-0.736)	0.716 (0.694-0.743)	0.723 (0.701-0.744)
*P* value for test of equality for AUCs^c^	<.001	.29	.07	<.001

Abbreviations: AUC, area under the receiver operating characteristic curve; CRP, C-reactive protein.

^a^ Compared with the Walter Index plus delirium, the test of equality for AUCs had a *P* value of .10.

^b^ Optimism is defined as the mean, over 100 datasets, of the difference between the performance of the model on bootstrap samples and the original dataset.

^c^ Compared with the external validation of the Walter Index alone (AUC, 0.714; 95% CI, 0.691-0.736).

The Charlson Comorbidity Index demonstrated lower discrimination than the Walter Index for predicting 12-month mortality (AUC, 0.640; 95% CI, 0.616-0.664; *P* < .001). The Walter Index also proved to be more clinically useful for making prognosis-based decisions than the Charlson Comorbidity Index. From a risk threshold of 0.09 onward, the Walter Index showed a higher net benefit compared with the Charlson Comorbidity Index, with this advantage generally maintained across increasing thresholds (eFigure 2 and eTable 2 in Supplement 1).

## Discussion

Our study examined the predictive ability of the Walter Index in a contemporary Brazilian cohort, exploring its external validity and transportability. Our findings support the favorable discrimination and calibration of the Walter Index in predicting 12-month posthospitalization mortality among adults aged 70 years or older, with an AUC indicative of reliable discrimination. These results highlight the enduring relevance of the constituent parameters of the Walter Index across distinct settings. Moreover, our study offered a comprehensive exploration of the index’s applicability in a non-US setting while also investigating additional time points and mortality predictors.^15,18^

The first reports of use of the Walter Index outside the US came from Italy, focused on 6-month mortality, and demonstrated its ability to predict mortality in association with male sex, dependencies in activities of daily living, heart failure, and cancer.^33^ Studies in Spain also demonstrated the potential of the index to enhance care plans for older adults compared with other indexes, although these studies lacked detailed methodologic descriptions.^9,13^ A broader examination in Belgium confirmed the moderate predictive ability of the Walter Index but noted it performed similarly to other models.^14^ A recent validation in China showed unexpectedly high calibration and discrimination.^34^ Despite these studies’ generally favorable results, their limitations warranted more comprehensive external validations.

In the original Walter Index validation cohort,^7^ the mean age was 79 years, 61% of participants were female, and 88% were White. At discharge, 15% were fully dependent. The cohort in our study was slightly older, with a larger percentage of female participants and a smaller percentage of White participants; also, participants had greater functional dependency. When comparing 12-month mortality rates, the Brazilian cohort had numerically higher mortality than the original cohort^7^ in the lowest risk group and numerically lower mortality in the other 3 risk groups. Our findings advance the understanding of the instrument’s applicability across diverse geographic, health care, and cultural settings. Many prognostic tools are seldom reevaluated in different contexts, limiting their global applicability. In many countries where health care resources are scarce or primary care systems are overburdened, hospitalizations may provide one of the few opportunities for comprehensive health assessments. Hence, instruments like the Walter Index can be crucial for making informed decisions, such as determining when a palliative care approach may be more appropriate, thereby ensuring that patients receive the most suitable care possible even after discharge.

Although the Walter Index was developed using data from the 1990s,^7^ this evaluation using data from the 2010s within a public health system in a different country and language showed that the index largely maintained its predictive performance.^15^ By applying the index to a modern health care environment with dynamics significantly different from the original setting, we found in this study that the index’s utility transcended aspects of validation such as variations in time, geography, health care infrastructure, and social factors.^35^ One notable consideration is that implementing the Walter Index requires assessing ADL dependency at discharge, a data point that may not be universally applied in clinical practice. Our results indicate that incorporating functional assessments throughout the care process could improve prognostication and support better planning for patient outcomes.

The higher net benefit compared with the Charlson Comorbidity Index also means that the Walter Index was more effective in correctly identifying patients at risk of mortality while minimizing unnecessary interventions for those not at risk. In practical terms, the findings suggest that using the Walter Index to guide clinical decisions would lead to better patient outcomes by more accurately targeting those who would benefit from further prognosis-based interventions, thereby improving the overall efficiency and effectiveness of health care delivery. Adding delirium to the Walter Index marginally yet significantly improved 12-month mortality discrimination. This suggests that including delirium captured risks not accounted for in the original model. Conversely, other factors, such as CRP level and frailty, may have been redundant with the roles of albumin level and activities of daily living, respectively.

Importantly, although the index’s performance did not match the higher levels set in the original study^7^ (a result that was anticipated), no other tools to our knowledge have consistently surpassed its effectiveness.^1,14,34^ Furthermore, enhancing the index with new predictors, such as delirium, could offer an avenue for improvement. Based on our results, we have revised ePrognosis to include delirium as a risk factor in the Portuguese version of the Walter Index, reflecting our commitment to improving predictive accuracy and clinical utility across settings.

### Strengths and Limitations

Our study has important strengths. First, its large sample size and comprehensive data collection ensured a detailed and robust analysis of the applicability of the Walter Index in a non-US setting. We also addressed most components of transportability, including geography, history, methods, and follow-up. Unlike other studies,^9,11,12,13,14^ the inclusion of additional variables in our analysis demonstrated that revisiting prognostic models with fresh approaches and insight can improve predictive accuracy. Furthermore, we were able to retain low rates of loss to follow-up, especially for a cohort of older adults with frailty.

Our study’s limitations must also be acknowledged. First, since the study was conducted in a single tertiary public university hospital, our findings may not be generalizable to other health care settings within Brazil. While the sample was representative of Brazilian older adults hospitalized in tertiary public hospitals, these individuals may have had different demographics and higher levels of comorbidities and functional impairments compared with the overall population of hospitalized older adults. Second, the study’s inclusion criteria, restricted to patients aged 70 years or older with hospital stays of at least 2 days, may limit generalizability to younger patients or those with shorter hospitalizations. Third, while the AUCs were satisfactory overall, discrimination was better for short-term outcomes at lower risk levels and better for longer-term outcomes at higher risk levels. Fourth, as in the original study,^7^ ADLs were assessed at discharge, which is 1 snapshot in time. Functional abilities can fluctuate during the acute hospital stay, and transient dependencies may result from the acute illness or deconditioning. Future research should examine the potential added benefit of including preadmission ADLs in the Walter Index.

## Conclusions

Our study findings supported the usefulness of the Walter Index in predicting 12-month posthospitalization mortality among adults aged 70 years or older with a minimum hospital stay of 2 days within a Brazilian public health system setting, demonstrating good calibration and moderate discriminative power. The enduring relevance of the core components of the index emphasizes its potential for widespread application. Future research should prioritize continuous evaluation and adaptation of prognostic models, broadening their application to different settings and incorporating new variables that may impact outcomes.

## Notes

Supplement 1. **eTable 1.** Comparison of Walter Index Components From the Original Derivation Model and Brazilian Recalibrated Model **eTable 2.** Comparative Net Benefit at Different Risk Thresholds for the Walter Index and the Charlson Comorbidity Index in Predicting 12-Month Posthospitalization Mortality in Older Adults **eFigure 1.** Receiver Operating Characteristic Curves for Posthospitalization Mortality According to the Walter Index **eFigure 2.** Decision Curve Analysis of the Walter Index and Charlson Comorbidity Index for Predicting 12-Month Posthospitalization Mortality in Older Adults

Supplement 2.
Data Sharing Statement
