# Supplementary material for: External Validation of the Walter Index for Posthospitalization Mortality Prediction in Older Adults
Source: JAMA Netw Open. 2025 Jan 22;8(1):e2455475. doi: 10.1001/jamanetworkopen.2024.55475 (PMC11755200; doi:10.1001/jamanetworkopen.2024.55475)
Supplement: Supplement 1. — eTable 1. Comparison of Walter Index Components From the Original Derivation Model and Brazilian Recalibrated Model eTable 2. Comparative Net Benefit at Different Risk Thresholds for the Walter Index and the Charlson Comorbidity Index in Predicting 12-Month Posthospitalization Mortality in Older Adults eFigure 1. Receiver Operating Characteristic Curves for Posthospitalization Mortality According to the Walter Index eFigure 2. Decision Curve Analysis of the Walter Index and Charlson Comorbidity Index for Predicting 12-Month Posthospitalization Mortality in Older Adults [file jamanetwopen-e2455475-s001.pdf]

Supplementary Online Content

Avelino-Silva TJ, Lee SJ, Covinsky KE, et al. External validation of the Walter Index for posthospitalization mortality prediction in older adults. *JAMA Netw Open*. 2025;8(1):e2455475. doi:10.1001/jamanetworkopen.2024.55475

eTable 1. Comparison of Walter Index Components From the Original Derivation Model and Brazilian Recalibrated Model
eTable 2. Comparative Net Benefit at Different Risk Thresholds for the Walter Index and the Charlson Comorbidity Index in Predicting 12-Month Posthospitalization Mortality in Older Adults
eFigure 1. Receiver Operating Characteristic Curves for Posthospitalization Mortality According to the Walter Index
eFigure 2. Decision Curve Analysis of the Walter Index and Charlson Comorbidity Index for Predicting 12-Month Posthospitalization Mortality in Older Adults

This supplementary material has been provided by the authors to give readers additional information about their work.

© 2025 Avelino-Silva TJ et al. *JAMA Network Open*.

eTable 1. Comparison of Walter Index components from the original derivation model and Brazilian recalibrated model.

Risk Factor	Walter Index (2001) Adjusted Coef. (95%CI)	Adjusted OR (95%CI)	p-value	Points	Walter Index (External validation) Adjusted Coef. (95%CI)	Adjusted OR (95%CI)	p-value	Points
Male sex	NA	1.4 (1.1-1.8)	0.01	1	0.337 (0.127, 0.547)	1.40 (1.14-1.73)	0.002	2
ADL dependencies at discharge								
Dependent in 1-4 ADLs	NA	2.1 (1.6-2.8)	<0.001	2	0.678 (0.422, 0.933)	1.97 (1.53-2.54)	<0.001	3
Dependent in all ADLs	NA	5.7 (4.2-7.7)	<0.001	5	1.219 (0.956-1.483)	3.39 (2.60-4.410	<0.001	6
Comorbid conditions								
Congestive heart failure	NA	2.0 (1.5-2.5)	<0.001	2	0.210 (0.101, 0.319)	1.23 (1.11-1.38)	<0.001	1
Solitary cancer	NA	2.6 (1.7-3.9)	<0.001	3	0.264 (-0.020, 0.548)	1.20 (0.98-1.73)	0.07	0
Metastatic cancer	NA	13.4 (6.2-29)	<0.001	8	1.680 (1.236, 2.125)	5.37 (3.44-8.37)	<0.001	8
Laboratory values on admission								
Creatinine, mg/dL >3.0	NA	1.7 (1.2-2.5)	0.01	2	0.324 (0.051, 0.596)	1.38 (1.05-1.82)	0.02	2
Albumin, g/dL 3.0-3.4	NA	1.7 (1.2-2.3)	0.001	1	0.415 (0.175, 0.656)	1.52 (1.19-1.93)	0.001	2
Albumin, g/dL <3.0	NA	2.1 (1.4-3.0)	<0.001	2	0.676 (0.425, 0.928)	1.97 (1.53-2.53)	<0.001	3
Intercept	NA				-2.482 (-2.708, -2.256)	0.08 (0.07-0.10)	<0.001	

Coef.: coefficient; 95%CI: 95% confidence interval; OR: odds ratio; ADL: activities of daily living; NA: not available.

© 2025 Avelino-Silva TJ et al. *JAMA Network Open*.

eTable 2. Comparative net benefit at different risk thresholds for the Walter Index and the Charlson Comorbidity Index in predicting 12-month posthospitalization mortality in older adults.

Risk threshold	Treat all net benefit[a]	Treat none net benefit	Charlson net benefit	Walter Index net benefit
0.01	0.2246203	0	0.2246203	0.2246203
0.02	0.2167083	0	0.2167083	0.2167083
0.03	0.2086331	0	0.2086331	0.2086331
0.04	0.2003897	0	0.2003897	0.2003897
0.05	0.1919727	0	0.1919727	0.1919727
0.06	0.1833767	0	0.1833767	0.1833767
0.07	0.1745958	0	0.1745958	0.1745958
0.08	0.165624	0	0.165624	0.165624
0.09	0.1564551	0	0.1564551	0.1624595
0.1	0.1470823	0	0.1470823	0.1547162
0.11	0.137499	0	0.137499	0.1466898
0.12	0.1276978	0	0.1276978	0.1395847
0.13	0.1176714	0	0.1221078	0.1323162
0.14	0.1074117	0	0.1132424	0.1240673
0.15	0.0969107	0	0.1063267	0.1178375
0.16	0.0861596	0	0.0975505	0.1114594
0.17	0.0751495	0	0.0885629	0.1026003
0.18	0.0638709	0	0.0773381	0.097368
0.19	0.0523137	0	0.0708988	0.0920064
0.2	0.0404676	0	0.0642986	0.0863309
0.21	0.0283216	0	0.0582278	0.0817548
0.22	0.0158642	0	0.0531083	0.0770614
0.23	0.0030832	0	0.0478557	0.0722461
0.24	-0.0100341	0	0.0415373	0.0690837
0.25	-0.0235012	0	0.0377698	0.0654676
0.26	-0.0373323	0	0.0339004	0.0617538
0.27	-0.0515423	0	0.0273381	0.0579383
0.28	-0.0661471	0	0.0248401	0.0540168
0.29	-0.0811632	0	0.0222718	0.0477505
0.3	-0.0966084	0	0.01963	0.044964
0.31	-0.1125013	0	0.0158847	0.0420968
0.32	-0.1288616	0	0.0141981	0.0391452
0.33	-0.1457103	0	0.0124611	0.0361054
0.34	-0.1630695	0	0.0106715	0.0323632
0.35	-0.1809629	0	0.0143331	0.0304095
0.36	-0.1994155	0	0.0131745	0.0283948
0.37	-0.2184538	0	0.011979	0.0263161
0.38	-0.2381063	0	0.010745	0.0241703
0.39	-0.2584031	0	0.0094705	0.0226206
0.4	-0.2793765	0	0.01247	0.0213429
0.41	-0.3010609	0	0.01184	0.0200219
0.42	-0.3234929	0	0.0111883	0.0186554
0.43	-0.3467121	0	0.0105137	0.0172409
0.44	-0.3707605	0	0.009815	0.015776
0.45	-0.3956835	0	0.0055919	0.0163506
0.46	-0.4215294	0	0.0051559	0.0154543
0.47	-0.4483508	0	0.0047034	0.0145242
0.48	-0.4762037	0	0.0042335	0.0135584
0.49	-0.5051488	0	0.002123	0.0125547
0.5	-0.5352518	0	0.0017986	0.0125899
0.51	-0.5665835	0	0.0014609	0.0120467

© 2025 Avelino-Silva TJ et al. *JAMA Network Open*.

0.52	-0.5992206	0	0.0011091	0.0114808
0.53	-0.6332466	0	0.0007424	0.0108909
0.54	-0.668752	0	-0.0014701	0.0102753
0.55	-0.7058353	0	-0.0016787	0.0096323
0.56	-0.7446043	0	-0.0018967	0.0065075
0.57	-0.7851765	0	-0.0021248	0.0059562
0.58	-0.8276807	0	-0.0023638	0.0053786
0.59	-0.8722583	0	-0.0035796	0.0047728
0.6	-0.9190648	0	-0.003777	0.0041367
0.61	-0.9682716	0	-0.0039845	0.003468
0.62	-1.020068	0	-0.004203	0.0024233
0.63	-1.074665	0	-0.0020902	0.0018861
0.64	-1.132294	0	-0.0021982	0.0013189
0.65	-1.193217	0	-0.0023124	0.0007194
0.66	-1.257723	0	-0.0024333	0.0000846
0.67	-1.326139	0	-0.0011009	-0.000545
0.68	-1.398831	0	-0.0011691	-0.0011241
0.69	-1.476213	0	-0.0012416	-0.0017405
0.7	-1.558753	0	-0.0013189	-0.0023981
0.71	-1.646986	0	-0.0014016	-0.003101
0.72	-1.741521	0	-0.0014902	-0.0019013
0.73	-1.843059	0	-0.0015854	-0.0024247
0.74	-1.952407	0	-0.0016879	-0.0029884
0.75	-2.070504	0	-0.0021583	-0.0035971
0.76	-2.198441	0	-0.0022782	-0.0042566
0.77	-2.337504	0	-0.0024085	-0.0051924
0.78	-2.489209	0	-0.0025507	-0.00569
0.79	-2.655361	0	-0.0027064	-0.006235
0.8	-2.83813	0	-0.0028777	-0.0068345
0.81	-3.040136	0	-0.003067	-0.0038811
0.82	-3.264588	0	0	-0.0041966
0.83	-3.515446	0	0	-0.0045493
0.84	-3.797662	0	0	-0.0034173
0.85	-4.117506	0	0	-0.003717
0.86	-4.483042	0	0	-0.0040596
0.87	-4.904815	0	0	-0.0024073
0.88	-5.396883	0	0	-0.0026379
0.89	-5.978417	0	0	-0.0029104
0.9	-6.676259	0	0	-0.0032374
0.91	-7.529177	0	0	-0.0036371
0.92	-8.595324	0	0	0
0.93	-9.966084	0	0	0
0.94	-11.79377	0	0	0
0.95	-14.35252	0	0	0
0.96	-18.19065	0	0	0
0.97	-24.58753	0	0	0
0.98	-37.38129	0	0	0
0.99	-75.76259	0	0	0

[a] Net benefit is defined as the proportion of true positives minus the proportion of false positives weighted by the risk threshold. The Walter Index demonstrates a higher net benefit compared to the Charlson Comorbidity Index, particularly at thresholds greater than 0.09, indicating superior performance in identifying at-risk patients for 12-month post-hospital mortality.

© 2025 Avelino-Silva TJ et al. *JAMA Network Open*.

eFigure 1. Receiver operating characteristic curves for posthospitalization mortality according to the Walter Index.

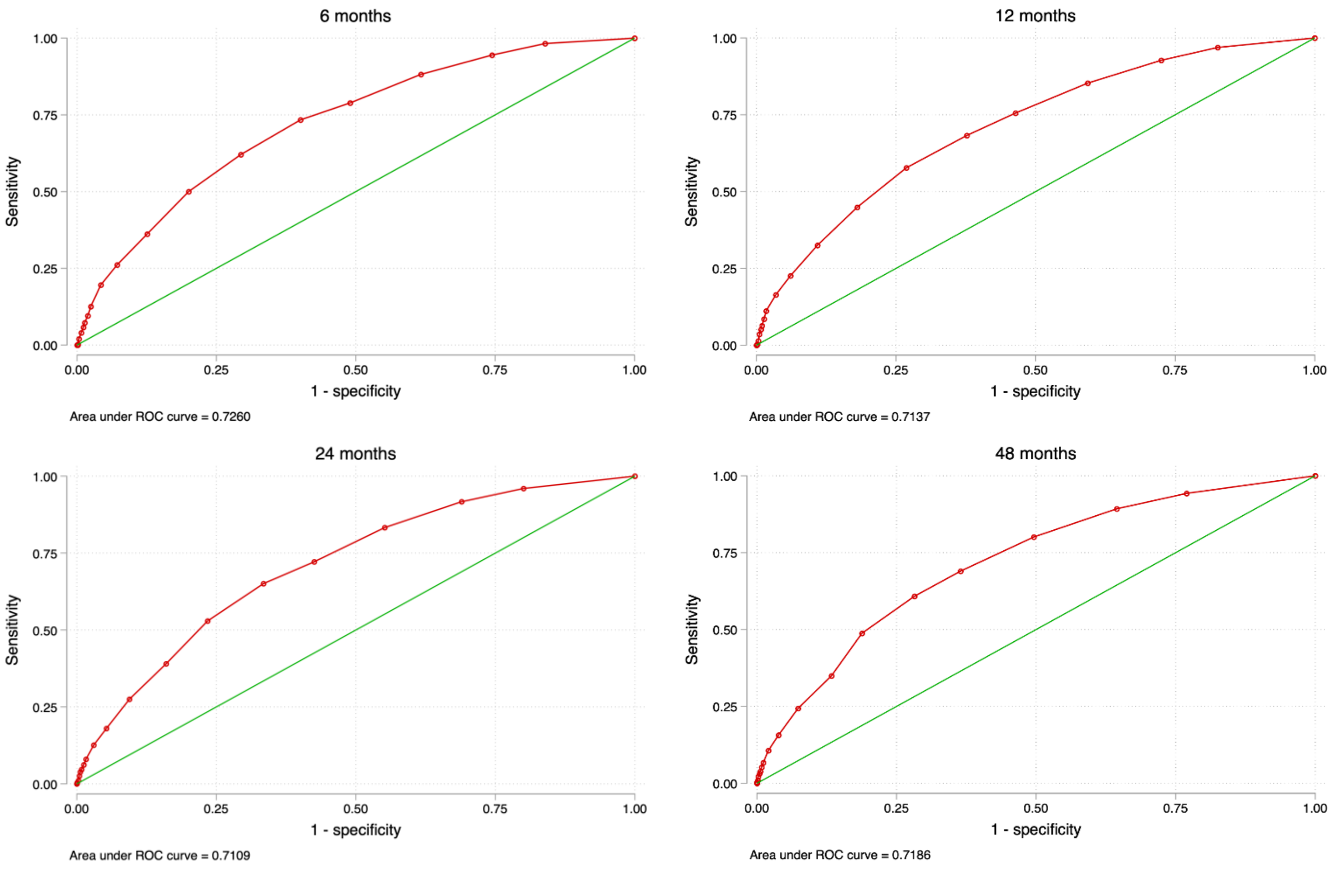

© 2025 Avelino-Silva TJ et al. *JAMA Network Open*.

eFigure 2: Decision curve analysis of the Walter Index and Charlson Comorbidity Index for predicting 12-month posthospitalization mortality in older adults.

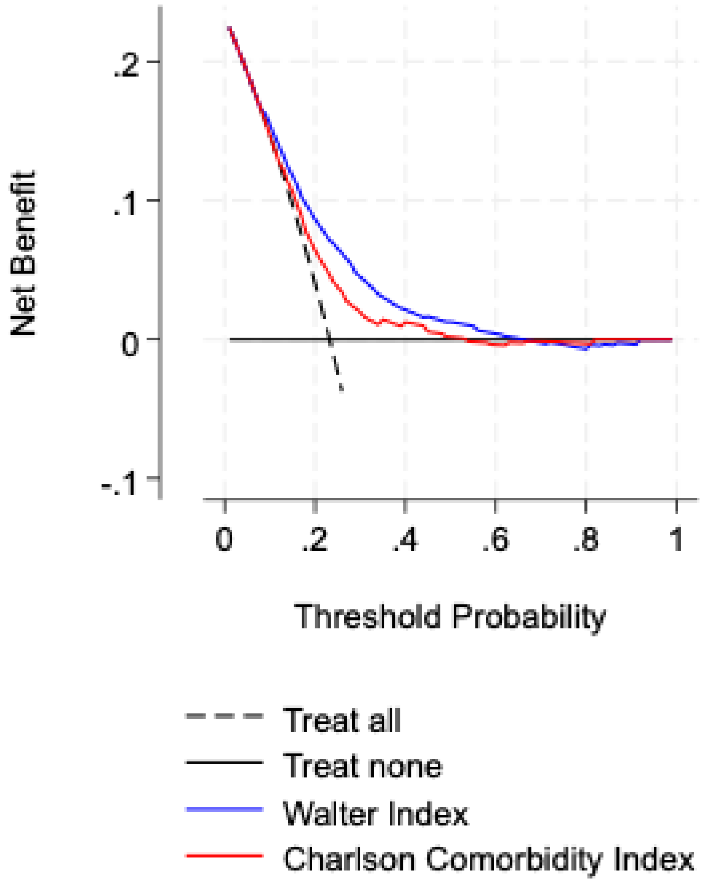

At lower thresholds (e.g., 0.01 to 0.08), the net benefits of using both the Charlson Comorbidity Index and the Walter Index are similar and consistently higher than considering no patients at risk. However, at thresholds of 0.09 and above, the Walter Index shows an increased net benefit compared to the Charlson Comorbidity Index, with this higher net benefit generally maintained across increasing thresholds (0.1 to 0.95), indicating better performance in identifying at-risk patients. For example, at a 10% threshold, the net benefit for the Walter Index is 0.1547 compared to 0.1471 for the Charlson Comorbidity Index; at a 30% threshold, the net benefit is 0.0449 for the Walter Index versus 0.0196 for the Charlson Comorbidity Index; and at a 50% threshold, the net benefit is 0.0126 for the Walter Index versus 0.0018 for the Charlson Comorbidity Index (eTable 2).

© 2025 Avelino-Silva TJ et al. *JAMA Network Open*.
