# Supplementary material for: External Validation of the Walter Index for Posthospitalization Mortality Prediction in Older Adults
Source: JAMA Netw Open. 2025 Jan 22;8(1):e2455475. doi: 10.1001/jamanetworkopen.2024.55475 (PMC11755200; doi:10.1001/jamanetworkopen.2024.55475)
Supplement: Supplement 2. — Data Sharing Statement [file jamanetwopen-e2455475-s002.pdf]

Data Sharing Statement

Avelino-Silva. External Validation of the Walter Index for Posthospitalization Mortality Prediction in Older Adults. *JAMA Netw Open*. Published January 22, 2025. doi:10.1001/jamanetworkopen.2024.55475

Data
Data available: No
